# Effects of Mindfulness-Based Cognitive Therapy on Body Awareness in Patients with Chronic Pain and Comorbid Depression

**DOI:** 10.3389/fpsyg.2016.00967

**Published:** 2016-06-30

**Authors:** Marasha de Jong, Sara W. Lazar, Kiran Hug, Wolf E. Mehling, Britta K. Hölzel, Alexander T. Sack, Frenk Peeters, Heidi Ashih, David Mischoulon, Tim Gard

**Affiliations:** ^1^Depression Clinical and Research Program, Massachusetts General Hospital, Harvard Medical SchoolBoston, MA, USA; ^2^Department of Psychiatry and Psychology, School of Mental Health and Neuroscience, Maastricht University Medical CenterMaastricht, Netherlands; ^3^MondriaanMaastricht, Netherlands; ^4^Department of Psychiatry, Massachusetts General Hospital, Harvard Medical SchoolBoston, MA, USA; ^5^Department of Psychosomatic Medicine, Medical Faculty, Medical Center-University of FreiburgFreiburg, Germany; ^6^Division of Clinical Psychology and Psychotherapy, Faculty of Psychology, University of BaselBasel, Switzerland; ^7^Department of Family and Community Medicine, Osher Center for Integrative Medicine, University of California, San FranciscoSan Francisco, CA, USA; ^8^Department of Neuroradiology, Klinikum rechts der Isar, Technical University of MunichMunich, Germany; ^9^Department of Cognitive Neuroscience, Faculty of Psychology and Neuroscience, Maastricht UniversityMaastricht, Netherlands; ^10^Institute for Complementary and Integrative Medicine, University Hospital Zurich, University of ZurichZurich, Switzerland; ^11^Translational Neuromodeling Unit, Institute for Biomedical Engineering, University of Zurich and Swiss Federal Institute of Technology (ETH)Zurich, Switzerland

**Keywords:** mindfulness-based cognitive therapy, mindfulness meditation, interoceptive awareness, body awareness, pain catastrophizing, chronic pain, depression, mediation

## Abstract

Body awareness has been proposed as one of the major mechanisms of mindfulness interventions, and it has been shown that chronic pain and depression are associated with decreased levels of body awareness. We investigated the effect of Mindfulness-Based Cognitive Therapy (MBCT) on body awareness in patients with chronic pain and comorbid active depression compared to treatment as usual (TAU; *N* = 31). Body awareness was measured by a subset of the Multidimensional Assessment of Interoceptive Awareness (MAIA) scales deemed most relevant for the population. These included: Noticing, Not-Distracting, Attention Regulation, Emotional Awareness, and Self-Regulation. In addition, pain catastrophizing was measured by the Pain Catastrophizing Scale (PCS). These scales had adequate to high internal consistency in the current sample. Depression severity was measured by the Quick Inventory of Depressive Symptomatology—Clinician rated (QIDS-C_16_). Increases in the MBCT group were significantly greater than in the TAU group on the “Self-Regulation” and “Not Distracting” scales. Furthermore, the positive effect of MBCT on depression severity was mediated by “Not Distracting.” These findings provide preliminary evidence that a mindfulness-based intervention may increase facets of body awareness as assessed with the MAIA in a population of pain patients with depression. Furthermore, they are consistent with a long hypothesized mechanism for mindfulness and emphasize the clinical relevance of body awareness.

## Introduction

Chronic pain is a highly prevalent and disabling condition with major impact on individuals, their significant others, and society (Turk et al., 2011). Prevalence rates for chronic pain range from 10 to 30% (Reid et al., 2011), and Major Depressive Disorder (MDD) is the most frequent psychiatric disorder in patients with chronic pain, with a 12-month prevalence ranging from 18% in population based settings up to 85% in specialized pain clinics (Bair et al., 2003). Since patients who suffer from both chronic pain and depression are particularly difficult to treat (Tunks, 2008), more effective interventions for this population are needed.

Mindfulness-based interventions have recently been shown to be effective for the treatment of chronic pain with small to moderate effect sizes on pain and depression (Veehof et al., 2011). Mindfulness-based therapies, and particularly mindfulness based cognitive therapy (MBCT), also have been shown to be effective for relapse prevention in recurrent depression and the treatment of active depression (Hofmann et al., 2010; Piet and Hougaard, 2011; Marchand, 2012; Sipe and Eisendrath, 2012). Results from a recent pilot randomized controlled trial (RCT) suggested that MBCT may be an effective intervention for the treatment of active depression in a population with chronic pain (de Jong et al., in press).

Because mindfulness-based interventions seem beneficial for chronic pain and depression, the question arises how mindfulness exerts its effects. Mindfulness entails paying attention to present moment experience, including thoughts, emotions, and bodily sensations. Training body awareness is a significant component of most mindfulness-based interventions, including the body scan, in which individuals specifically pay attention to all parts of the body; and yoga, which entails paying attention to movements of the body (Kabat-Zinn, 1990; Segal et al., 2013). Body awareness has been proposed as a potential mechanism for the therapeutic effects of mindfulness and is considered an integral part of the mindfulness construct (Mehling et al., 2009; Hölzel et al., 2011; Farb et al., 2015). The definition of body awareness emphasizes the fact that this is a complex multi-dimensional construct: “the sensory awareness that originates from the body's physiological states, processes (including pain and emotion), and actions (including movement), and functions as an interactive process that includes a person's appraisal and is shaped by attitudes, beliefs, and experience in their social and cultural context” (Mehling et al., 2012). In this article, the terms body awareness and interoceptive awareness are used interchangeably.

Several studies lend support to the notion of enhanced body awareness through mindfulness training. For example, meditators have been reported to show greater coherence between objective physiological data and their subjective experience—in regard to both emotional experience (Sze et al., 2010) and sensitivity of body regions (Fox et al., 2012). With regard to the heart beat perception task, which assesses the ability of subjects to accurately determine their heartbeat rate by comparing the subjectively counted heartbeats to heartbeats measured by an electrocardiogram, a number of studies with small sample sizes did not find increased interoceptive accuracy in meditators (Nielsen and Kaszniak, 2006; Khalsa et al., 2008; Melloni et al., 2013; Parkin et al., 2014). However, a large (*N* = 160), recent longitudinal study revealed that heart beat accuracy was increased after 39 weeks of a mindfulness-based contemplative intervention (Bornemann and Singer, 2015). Neuroimaging studies indicate mindfulness training-related changes in brain function and structure in regions that are thought to be involved in body awareness (Lazar et al., 2005; Hölzel et al., 2008; Farb et al., 2010, 2013; Gard et al., 2012).

It has been long postulated that interoceptive awareness plays an important role in the experience of emotions (James, 1984) and there is empirical evidence that the extent to which one can accurately perceive bodily functions has a positive relationship with the intensity of emotions (Herbert et al., 2007). Former studies on this topic in clinical populations have mainly focused on anxiety disorders, which demonstrated a close association with increased interoceptive awareness (Ehlers and Breuer, 1996). Depression often entails anhedonia and blunted emotions. In fact, body awareness has been found to be reduced in individuals with depression (Ehlers and Breuer, 1992; Dunn et al., 2007), and higher levels of depressive symptoms are associated with decreased body awareness in healthy subjects (Pollatos et al., 2009). Reduction in interoceptive awareness in depression is also supported by a recent neuro-imaging study, which shows reduced effective connectivity in networks involved in interoception in patients with melancholia (Hyett et al., 2015). A recent study revealed that body awareness therapy resulted in decreased self-rated depressive symptoms, but no changes in body awareness were found (Danielsson et al., 2014). Whether improvements in body awareness lead to reduced depression has yet to be established.

Variations in body awareness appear to be particularly important in patients with chronic pain. Mehling et al. (2013), for example, reported differences in some dimensions of interoceptive awareness between patients with current or past low back pain and mind-body trained individuals. Neuro-scientific evidence indicates that some of the brain regions activated during pain are also activated when engaging in interoceptive awareness (Craig, 2003). Attention styles toward chronic pain sensations are of key importance for psychological pain management (Johnston et al., 2012), and fMRI studies suggest that mindfulness meditation facilitates a reduction of pain through increased sensory processing (Gard et al., 2012). Thus, body awareness and pain perception are closely linked on a neuro-biological level, such that the enhancement of specific styles or dimensions of body awareness may facilitate the self-regulation of pain.

Although body awareness is considered an integral part of the mindfulness construct, there has been a paucity of instruments that measure body awareness (Mehling et al., 2009). Previous body awareness questionnaires either measured non-adaptive forms of body awareness (as indicated in disorders such as panic disorder), were uni-dimensional, lacked systematic development, or did not measure body awareness specifically, but rather a more general observation ability (Mehling et al., 2012). The Multidimensional Assessment of Interoceptive Awareness (MAIA; Mehling et al., 2012) scale is a relatively new, multifaceted body awareness questionnaire that intends to fill the apparent gap.

Bornemann et al. (2015), who recently translated the MAIA into German and demonstrated good scale properties, found that a 3-month contemplative training that included the bodyscan and breath meditation techniques lead to changes on several scales in a sample of individuals with good psychological and physical health. Values on all scales increased following the training, and changes were significantly greater compared to a retest control group for most of the scales.

While there is evidence for an effect of a mind-body intervention on body awareness as measured with the MAIA in a healthy sample, no intervention studies have been reported on effects in chronic pain patients or in depressed individuals. In the present pilot RCT we investigated the longitudinal effects of MBCT on body awareness, as measured by the MAIA in a population of patients with chronic pain and comorbid depression. It was hypothesized that MBCT enhances aspects of body awareness in this population. Furthermore, as this is the first study using the MAIA in a sample of patients with chronic pain and comorbid active depression, we investigated the reliability of the MAIA scales in this population.

## Methods

### Participants

Participants in this add-on study were part of a larger clinical trial reported elsewhere (de Jong et al., in press; clinicaltrials.gov id: NCT01473615). Patients were recruited from different outpatient clinics through introduction of the study by their physicians, as well as via web-based advertisements and through several online mailing lists. After phone screening and a subsequent in-person screening visit, eligible participants were offered enrollment and were randomly assigned to treatment as usual (TAU) or TAU plus mindfulness-based cognitive therapy (MBCT). English-language literate individuals aged 18 or older were eligible if they (a) had persistent chronic pain for a minimum of 3 months; (b) met the DSM-IV criteria for MDD, Dysthymic Disorder, or Depressive disorder Not Otherwise Specified (NOS); and (c) a score ≥10 on the QIDS-C_16_ scale. After initiating the study the cutoff was reduced to a QIDS-C_16_ score ≥6 (indicative of at least mild depressive symptoms) to allow more ample recruitment.

Exclusion criteria were: (a) serious suicide or homicide risk; (b) current or past bipolar disorder, current psychotic symptoms, or a current or past primary psychotic disorder; (c) diagnosis of substance abuse or dependence disorder during the last 3 months; (d) general condition that impedes attendance in group interventions, such as severe personality disorders, cognitive impairment, or tendencies toward physical aggression; (e) severe and unstable medical illness including cardiovascular, hepatic, renal, respiratory, endocrine, neurological, or hematological disease; and (f) significant present meditation practice with more than 3 h of mindfulness, insight, or vipassana meditation per week. Patients were requested to keep their psychological and pharmacological treatment as stable as possible from 8 weeks before the beginning of the study until its conclusion.

**Figure 1:**
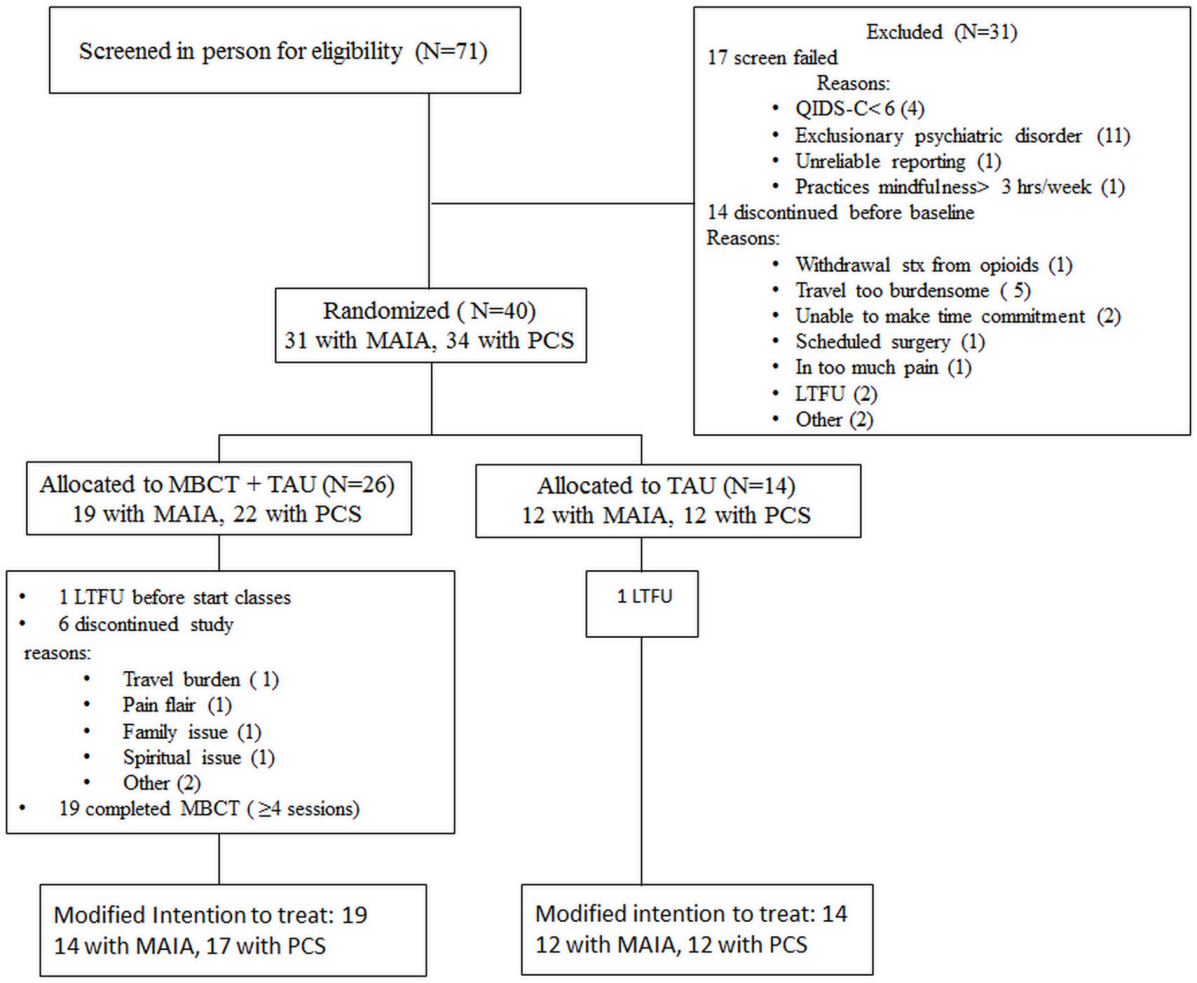
**Patient flow**. LTFU, lost to follow up; MAIA, Multidimensional Assessment of Interoceptive Awareness; MBCT, Mindfulness Based Cognitive Therapy; PCS, Pain Catastrophizing Scale; QIDS-C, Quick Inventory of Depressive Symptoms-Clinician Rated; TAU, Treatment As Usual.

Seventy-one participants were screened, of which 40 were randomized to TAU or MBCT + TAU in a 1:2 ratio (Figure 1). Participants received $40 for completed study participation and provided written informed consent. The study was approved by the Partners Human Research Committee, Massachusetts General Hospital (protocol 2011-P-001699/1).

### Measures

#### Multidimensional assessment of interoceptive awareness (MAIA)

The MAIA is a 32-item instrument that assesses body awareness on 6-point Likert-type scales that range from 0 (Never) to 5 (Always). It comprises eight scales, namely Noticing, Not-Distracting, Not-Worrying, Attention Regulation, Emotional Awareness, Self-Regulation, Body Listening, and Trusting (Mehling et al., 2012). The eight scales have been shown to have adequate to excellent internal-consistency reliabilities, with Cronbach's alphas from 0.66 to 0.87, and above 0.70 for five of the eight scales (Mehling et al., 2012). Because the current study was an add-on to a larger study, subject burden had to be kept to a minimum. For this reason only the scales deemed most relevant for the specific population were administered, namely Noticing, Not-Distracting, Attention Regulation, Emotional Awareness, and Self-Regulation.

The Noticing scale assesses the awareness of comfortable, neutral, and uncomfortable body sensations. Not-Distracting refers to not ignoring or distracting oneself from uncomfortable body sensations such as pain. Attention Regulation is the ability to maintain and regulate attention to body sensations, and Emotional Awareness is defined as consciousness of the interrelation of emotions and body sensations. Self-Regulation refers to the ability to control psychological distress by consciously attending to body sensations (Mehling et al., 2013). The MAIA Not Worrying scale assesses worrying or feeling emotionally distressed in response to uncomfortable body sensations including pain and was not administered, since its items are similar to the Pain Catastrophizing Scale, which was included in the larger study.

#### Pain catastrophizing scale (PCS)

The PCS is a 13-item scale, comprised of three subscales, that measures pain catastrophizing. Pain catastrophizing is defined as “an exaggerated negative mental set brought to bear during (actual or anticipated) painful experience” (Sullivan et al., 2001). The PCS total score can range from 0 to 52 and has been shown to have excellent reliability (Cronbach's alpha = 0.87; Sullivan et al., 2001).

#### Quick inventory of depressive symptomatology—clinician rated (QIDS-C_16_)

The QIDS-C is a widely used clinician rated instrument to assess depression symptom severity. The instrument is comprised of 16 items, has good psychometric properties, score range from 0 to 27 and is sensitive to changes in depressive severity (Rush et al., 2003; Trivedi et al., 2004).

### Procedure

Eligible participants were randomly assigned to TAU (control group) or MBCT + TAU (intervention group) in a 1:2 ratio, which allowed the main project to fill the MBCT groups with participants more quickly. An independent researcher not involved in the project generated the randomization sequence in blocks of five (using the sequence generator on www.random.org). In order to assure equal gender distribution in both groups we stratified for gender. The intervention group received an 8-week MBCT group skills program. Interoceptive awareness, pain catastrophizing, and depression were assessed at baseline (week 0), midpoint (week 4), and endpoint (week 8).

### Intervention

The intervention consisted of an 8-week group skills program with one 2-h mindfulness training session each week and individual exercises for homework practice. It was modeled on the MBCT program developed by Segal et al. (2013), which was developed as a program to address recurrent depressive episodes and combines elements of cognitive behavioral therapy (CBT), such as psycho-education, with experiential mindfulness practices. The program is intended to teach and foster a non-judgmental, accepting attitude toward one's internal, and external experience. For the current study the original program was adapted to our specific population by modifying the psychoeducation and CBT elements to a depressed CP population. This included psycho-education linking CP, negative thoughts, negative emotions, and depressive behaviors such as withdrawal; identifying automatic thoughts related to CP; and paying attention to behavioral elements such as pacing of activities. We also included meditations that specifically focused on cultivating mindfulness in relationship to CP. The MBCT program was led by two instructors, an experienced licensed independent clinical social worker (LICSW) and a fellow in psychology and was provided free of charge. Subjects that were assigned to TAU were waitlisted and offered the MBCT treatment after completion of the study. TAU included all regular visits with the pain physician, psychiatrist, psychotherapist and prescribed pain and/or antidepressant medications.

### Statistical analyses

Differences in patient characteristics at baseline were assessed by performing independent-samples *t*-tests for continuous variables and Chi-square tests for categorical variables. To evaluate internal consistency/reliability, Cronbach's alphas were assessed. Inter-scale correlations were obtained for the MAIA scales and the PCS total score based on the data of all subjects at baseline. The effects of intervention/group (MBCT + TAU vs. TAU) and time (baseline vs. endpoint) on the dependent variables body awareness and pain catastrophizing were assessed by conducting repeated measures analysis of variance (rmANOVA), with time as repeated measure, treatment group as between-subjects factor, and MAIA scales and PCS as dependent variables. Assumptions of normality and homogeneity were met. Paired samples *t*-tests were conducted to compare baseline and endpoint scores on the MAIA scales and the PCS within each group. As a measure of effect size, Cohen's d was calculated for each pre-post change. Analyses were conducted according to a modified intention-to-treat (ITT) principle with the last observation carried forward (LOCF). When endpoint measures were missing, midpoint measures were imputed, and if midpoint data were missing, baseline data were used. Only participants who attended at least four of the eight classes were included in the analyses.

The main study, to which this study was added on, revealed a significant effect of MBCT (group by time interaction) on depression as measured with the Quick Inventory of Depressive Symptomatology-Clinician rated (QIDS-C_16_) (de Jong et al., in press). To explore if and how this effects is mediated by the MAIA, a multiple mediator model was tested. The model comprised group (MBCT + TAU vs. TAU) as independent variable, depression measured at week 8 as dependent variable and MAIA scales (measured at week 8) that revealed significant group by time interactions, as mediators. Depression and respective MAIA scales measured at baseline (week 0) were included as covariates. Mediation analyses were conducted with a macro by Preacher and Hayes (2008) that implements a bootstrapping procedure to create confidence intervals for partial and total indirect effects. For mediation analyses only participants who participated in at least 4 classes and who had week 0 and week 8 data available were included in the analyses and 10,000 bootstrap iterations were used. All analyses were conducted with SPSS 21 (SPSS Inc., Chicago, IL, USA).

## Results

### Participant characteristics

For the main study, 71 patients were screened, of which 40 were randomized to TAU (*n* = 14) or MBCT + TAU (*n* = 26) in a 1:2 ratio. Of those 40, 34 completed the pain catastrophizing scale (PCS) and 31 the MAIA at baseline. Of the 14 patients in the TAU, 12 had PCS and MAIA data and were included in modified ITT analyses. Of the 26 patients in MBCT + TAU, 22 had PCS and 19 MAIA data. Five of the patients with MAIA and PCS data participated in <4 sessions of MBCT and were excluded from further analyses, resulting in 17 patients with PCS data and 14 with MAIA data in the modified ITT analyses (Figure 1). For the mediation analyses 11 subjects with MAIA data were in MBCT + TAU and 7 in TAU.

Characteristics of participants (*N* = 29) who attended at least four classes (MBCT+TAU) or had four clinic visits (TAU), are shown in Table 1. Types of chronic pain included: chronic back pain, neuropathic pain, osteoarthritis, fibromyalgia, and migraines. There were no significant differences in demographics and patient characteristics (see Table 1) or in average baseline scores on the five MAIA scales or total PCS scores between the two groups at baseline (see Table 2). For the collapsed sample that completed the MAIA, regardless of class attendance, participants (*N* = 31) were on average 50 years old (*M* = 49.45, *SD* = 10.58), and college graduates (*M* = 16.30, *SD* = 2.55 years of education). Most of the participants were female (74.2%), Caucasian (90.3%), and non-Hispanic (83.9%). Three (9.7%) participants were African American and one (3.2%) was Hispanic. The largest proportion of participants was married (46.7%) or never married (36.7%), and five were separated or divorced (16.7%). Employed participants comprised 32.3% of the sample, and disabled ones comprised 32.3% of the sample. Most participants had (MDD; 83.9%), with the remaining 16.1% suffering from Depressive Disorder not otherwise specified (NOS). 46.4% of the participants were taking Anti-Depressant medication.

**Table 1:** **Participant characteristics (*N* = 29)**.

	**TAU**		**MBCT + TAU**		***t/χ^2^*-test**		
	***M/%***	***SD***	***M***	***SD***	***t/χ^2^***	***df***	***p***
Age (years)	51.67	10.08	50.06	11.68	0.39	27	0.703
Education (years)^a^	16.58	2.61	15.94	2.56	0.65	26	0.519
Gender (% female)	66.7		76.5		0.34	1	0.561
Race (%)					0.88	1	0.348
African-American	16.7		5.9				
Caucasian	83.3		94.1				
Ethnicity (%)					3.28	2	0.194
Hispanic	0.0		5.9				
Non-hispanic	100.0		76.5				
Unknown/Not reported			17.6				
Marital status (%)^a^					0.933	2	0.627
Never married	25.0		37.5				
Married/Live together	50.0		50.0				
Separated/Divorced	25.0		12.5				
Employment status (%)^a^					1.67	2	0.435
Employed	16.7		37.5				
Disabled	41.7		37.5				
Other/Not reported	41.7		25.0				
Type depression (%)					0.20	1	0.653
NOS	16.7		23.5				
MDD	83.3		76.5				
ADM (% taking)^b^	50.0		35.3		0.564	1	0.453

*ADM, Anti-Depressant Medication; MDD, Major Depressive Disorder; MBCT, Mindfulness Based Cognitive Therapy; NOS, Depressive Disorder Not Otherwise Specified; SD, Standard Deviation; TAU, Treatment As Usual*.

a *Based on N = 28 due to missing value*.

b *Based on N = 27 due to missing values*.

**Table 2:** **Baseline scores on MAIA and PCS**.

	**TAU**		**MBCT + TAU**		***t*-test**		
	***M***	***SD***	***M***	***SD***	***t***	***df***	***p***
**MAIA**							
Noticing	3.13	1.26	2.86	1.01	0.60	24	0.553
Attention regulation	2.08	1.34	2.37	1.05	0.60	24	0.550
Emotional awareness	2.65	1.63	2.71	1.29	0.11	24	0.912
Self-regulation	2.10	1.42	1.98	1.24	0.23	24	0.817
Not distracting	2.14	0.80	1.95	1.23	0.45	24	0.657
**PCS**	27.17	10.67	31.82	12.28	1.06	27	0.299

*MAIA, Multidimensional Assessment of Interoceptive Awareness; PCS, Pain Catastrophizing Scale*.

### Scale properties

Table 3 summarizes scale means with standard deviations, range of observed values, Cronbach's alphas, and range of item-scale correlations of the MAIA and the PCS for the entire sample (*N* = 31 and 34 respectively) at baseline. Cronbach's alphas for three of the five administered MAIA scales were excellent and ranged from 0.92 (Attention Regulation) to 0.94 (Emotional Awareness). The Not-Distracting and Noticing scales had alphas of 0.72 and 0.67 respectively. The PCS had a Cronbach's alpha of 0.93.

**Table 3:** **Scale properties (N)**.

**Scale**	**Number of items**	**Cronbach's Alpha**	**Range of item-scale correlations**	**Mean (SD)**	**Observed**	***N***
**MAIA**						
Noticing	4	0.67	0.65–0.74	3.10 (1.15)	1–5	31
Attention regulation	7	0.92	0.64–0.91	2.42 (1.22)	0.1–4.57	31
Emotional awareness	5	0.94	0.85–0.93	2.73 (1.50)	0–5	31
Self-regulation	4	0.93	0.89–0.96	1.92 (1.37)	0–4.5	31
Not distracting	3	0.72	0.49–0.84	2.04 (1.06)	0.3–5	31
**PCS**	13	0.93	0.51–0.85	30.18 (11.63)	4–52	34

*MAIA, Multidimensional Assessment of Interoceptive Awareness; PCS, Pain Catastrophizing Scale; SD, Standard Deviation*.

Table 4 shows Pearson correlations between the MAIA scales. The correlations ranged from 0.76 for Self-regulation and Emotional awareness and 0.71 for Emotional awareness and Noticing, to (*r* ≤ |0.29|) for Not Distracting, which did not correlate significantly with any other MAIA scale.

**Table 4:** **Scale-scale correlations**.

**Scale**	**Noticing**	**Attention regulation**	**Emotional awareness**	**Self- regulation**	**Not distracting**
Noticing	–				
Attention regulation	0.53^**^	–			
Emotional awareness	0.71^**^	0.62^**^	–		
Self-regulation	0.52^**^	0.50^**^	0.76^**^	–	
Not distracting	0.02	0.17	−0.29	−0.09	–

*Pearson product moment correlations among MAIA scales in the total sample (N = 31)*.

** *Correlations are significant at p < 0.01*.

*MAIA, Multidimensional Assessment of Interoceptive Awareness*.

### Effects of MBCT on body awareness

**Figure 2:**
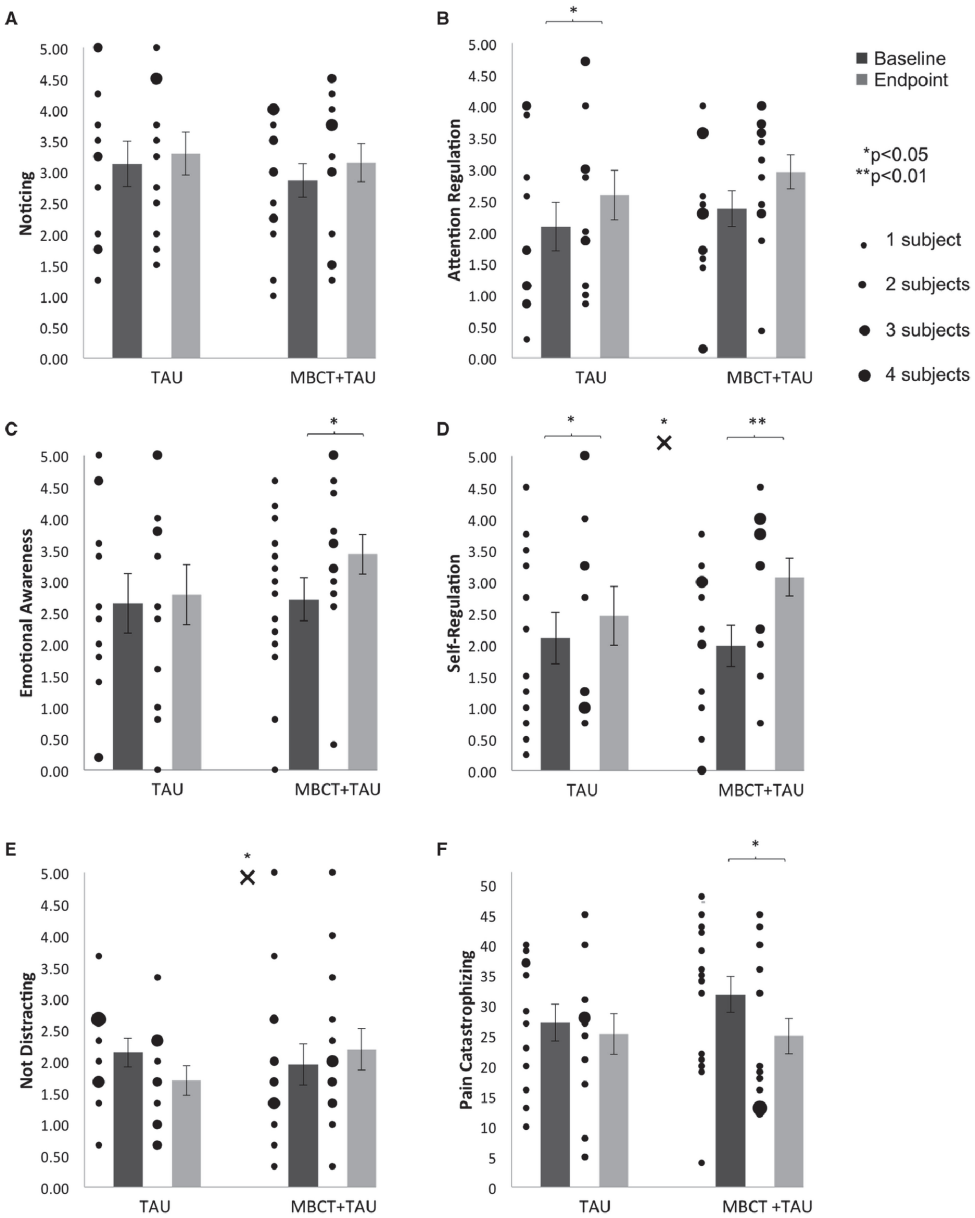
**Individual data and averages for baseline and endpoint**. **(A)** Noticing, **(B)** Attention Regulation, **(C)** Emotional Awareness, **(D)** Self-regulation, **(E)** Not Distracting, **(F)** Pain Catastrophizing. Error bars are ± 1 SEM; Asterisks above the × symbols indicate significant *p*-values based on group by time interaction effects as revealed by repeated measures ANOVAs; Asterisks above the bars indicate significant *p*-values based on pre-post treatment changes as revealed by paired-samples *t*-tests.

For Noticing, no significant group-by-time interaction [*F*_(1, 24)_ = 0.18, *p* = 0.676, $\eta_{p}^{2}$ = 0.007] and no significant main effect of time [*F*_(1, 24)_ = 2.59, *p* = 0.121, $\eta_{p}^{2}$ = 0.097] was found. No significant within group changes between the pre- and the post-treatment measurements were found in either group with effect sizes of the pre-post change being medium for the treatment group but small for the control group (Figure 2A, Table 5).

**Table 5:** **Changes in scores for body awareness and pain catastrophizing**.

	**TAU**						**MBCT + TAU**					
	**Pre *M (SD)***	**Post *M (SD)***	***t***	***df***	***p***	***d***	**Pre *M (SD)***	**Post *M (SD)***	***t***	***df***	***p***	***d***
**MAIA**												
Noticing	3.13 (1.26)	3.29 (1.20)	1.30	11	0.220	+0.134	2.86 (1.01)	3.14 (1.15)	1.21	13	0.247	+0.262
Attention regulation	2.08 (1.34)	2.58 (1.36)	2.52	11	0.029	+0.371	2.37 (1.05)	2.95 (1.01)	1.46	13	0.167	+0.564
Emotional awareness	2.65 (1.63)	2.78 (1.66)	0.73	11	0.482	+0.081	2.71 (1.29)	3.43 (1.19)	2.17	13	0.049	+0.573
Self-regulation	2.10 (1.42)	2.46 (1.62)	2.49	11	0.030	+0.216	1.98 (1.24)	3.07 (1.12)	4.34	13	0.001	+0.913
Not distracting	2.14 (0.80)	1.69 (0.81)	−1.85	11	0.092	−0.553	1.95 (1.23)	2.19 (1.23)	1.20	13	0.253	+0.194
**PCS**	27.17 (10.67)	25.25 (11.52)	−0.57	11	0.580	−0.172	31.82 (12.28)	24.94 (12.16)	−2.23	16	0.041	−0.564

*Results of paired-samples t-tests and Cohen's d effect sizes for pre-post changes for treatment and control group*.

*MAIA, Multidimensional Assessment of Interoceptive Awareness; PCS, Pain Catastrophizing Scale; d, Cohen's d; SD, Standard Deviation*.

For the Attention Regulation scale, no significant group-by-time interaction effect [*F*_(1, 24)_ = 0.03, *p* = 0.863, $\eta_{p}^{2}$ ≤ 0.001] was found, but a significant main effect of time was revealed [*F*_(1, 24)_ = 5.34, *p* = 0.030, $\eta_{p}^{2}$ = 0.182]. Paired sample *t*-tests showed significant, medium size increases of Attention Regulation scores in the control group. The increase in Attention Regulation in the MBCT group did not reach statistical significance, despite its large effect size (Figure 2B, Table 5).

For Emotional Awareness, no significant group-by-time interaction effect [*F*_(1, 24)_ = 2.17, *p* = 0.153, $\eta_{p}^{2}$ = 0.083] was found, but a significant main effect of time was revealed [*F*_(1, 24)_ = 4.63, *p* = 0.042, $\eta_{p}^{2}$ = 0.162]. Paired samples *t*-tests revealed a large and significant increase in Emotional Awareness over time within the treatment group, while the change within the control group was small and not significant (Figure 2C, Table 5).

A rmANOVA revealed a significant group-by-time interaction for Self-Regulation [*F*_(1, 24)_ = 5.93, *p* = 0.023, $\eta_{p}^{2}$ = 0.198]. This interaction was driven by large and significant increases in Self-Regulation over time in the treatment group and small but significant increases in the control group. Furthermore, the analyses revealed a significant main effect of time [*F*_(1, 24)_ = 22.86, *p* < 0.001, $\eta_{p}^{2}$ = 0.488] (Figure 2D, Table 5).

For the Not Distracting scale, a significant group-by-time interaction was revealed [*F*_(1, 24)_ = 4.87, *p* = 0.037, $\eta_{p}^{2}$ = 0.169]. This interaction was driven by an increase in Not Distracting scores in the treatment group and a decrease within the control group, none of which reached statistical significance. No main effect of time [*F*_(1, 24)_ = 0.45, *p* = 0.511, $\eta_{p}^{2}$ = 0.018] was found (Figure 2E, Table 5).

For pain catastrophizing, no significant group-by-time interaction effect [*F*_(1, 27)_ = 1.15, *p* = 0.294, $\eta_{p}^{2}$ = 0.041] was revealed. The main effect of time approached significance [*F*_(1, 27)_ = 3.60, *p* = 0.069, $\eta_{p}^{2}$ = 0.118]. Analysis showed a large and significant decrease of pain catastrophizing within the MBCT group, but only a small and not significant decrease in the control group (Figure 2F, Table 5).

### Body awareness as mediator

**Figure 3:**
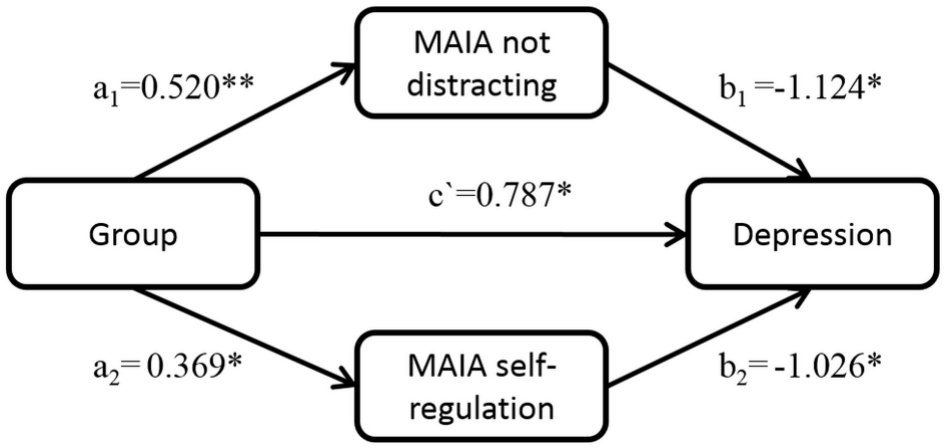
**Mediation model**. Mediation model for the effect of group [Mindfulness Based Cognitive Therapy + treatment as usual (TAU), vs. TAU alone] on depression (measured with the Quick Inventory of Depressive Symptomatology—Clinician rated) through the Not Distracting and Self-Regulation scales of body awareness as measured with the Multidimensional Assessment of Interoceptive Awareness (MAIA). Analyses are based on *N* = 18 and numbers are standardized regression coefficients. ^*^p < 0.05, ^**^p < 0.01.

A multiple mediator model as described by Preacher and Hayes (2008) was tested. The model (Figure 3) was comprised of group (MBCT + TAU vs. TAU) as independent variable, depression measured with QIDS-C_16_ at week 8 as dependent variable, and the MAIA Not Distracting and Self-Regulation scales, measured at week 8, as mediators. QIDS-C_16_, MAIA Not Distracting and Self-Regulation scales measured at week 0 were included as covariates.

Analyses resulting in bias-corrected confidence intervals (CI) based on 10,000 bootstrap iterations, revealed a significant indirect effect of group on depression through the MAIA scale Not Distracting (a_1_ × b_1_ = −3.584, 95% CI −8.880 to −0.357), but not through the Self-Regulation scale (a_2_ × b_2_ = −2.317, 95% CI −8.733 to 0.284). There also was a significant direct effect of group on depression (c′ = 4.817, *p* = 0.0485) independent of Self-Regulation or Not Distracting. These findings indicate that the effect of MBCT on depression was partially mediated by Not Distracting but not by Self-Regulation.

## Discussion

In the present study, we investigated the effects of MBCT on body awareness as measured with the MAIA in patients with chronic pain and comorbid active depression. To our knowledge, this is the first RCT that investigates the effects of MBCT on body awareness as measured with the MAIA in patients with chronic pain and depression. The MAIA appears a reliable instrument with scales of adequate consistency in this newly studied population. In accordance with our hypothesis, MBCT resulted in increases for several dimensions of body awareness in the studied patient population. More specifically, a significantly greater increase in Self-Regulation and Not Distracting, but no changes in Noticing in the MBCT group as compared to the TAU group were observed. In addition, participants in the MBCT group, but not in the control group, had increases in Emotional Awareness. For Pain Catastrophizing, we found significant decreases within the treatment group, but not within the control group. Furthermore, mediation analyses revealed that the effect of MBCT on depression was mediated by Not Distracting, but not by Self-Regulation. We discuss these results for each dimension of body awareness separately in more detail below.

### Self-regulation

The finding of a significantly greater increase in Self-Regulation ratings in the treatment than in the control group is in line with findings by Mehling et al. (2013, 2014). In two cross-sectional studies they showed that patients with chronic low back pain who were practicing mind-body therapies (Mehling et al., 2013), or yoga and meditation (Mehling et al., 2014) had greater self-regulatory body awareness than patients without such practice. In the cohort study (Mehling et al., 2014), this difference was more pronounced for the Self-Regulation dimension than for any of the other dimensions of body awareness. Similarly, Bornemann et al. (2015) also found that increases following 3 months of contemplative training including the bodyscan and breath awareness meditation were largest on the Self-Regulation scale (effect size *d* = 0.72). Our study extends the previous findings by demonstrating effects through a well-established intervention (MBCT) in a patient population (chronic pain with active depression).

The present findings of a change in Self-Regulation through a mindfulness intervention are consistent with a hypothesized link between mindfulness and enhanced self-management (Baer, 2003), a concept closely related to general self-regulation (Vohs and Baumeister, 2011). Our findings also converge with empirical evidence for better general self-regulation of chronic pain through a mindfulness intervention (Kabat-Zinn et al., 1985), as well as with evidence from paradigms with acute pain induction, in which mindfulness practice enhanced pain tolerance (Kingston et al., 2007; Gard et al., 2012), thereby having potential clinical implications.

### Emotional awareness

Paired samples *t*-tests revealed a large and significant increase in Emotional Awareness over time within the treatment group, while the change within the control group was small and not significant However, no significant group by time interaction was found. The significant increase in Emotional Awareness over time in the treatment group is in line with the expectation that the MBCT intervention increases the awareness of the connection between body sensations and emotional states and corresponds to results by Mehling et al. (2013, 2014). Their group found that levels of Emotional Awareness were higher in chronic pain patients with mind-body practice than in patients without such practice (Mehling et al., 2013). Bornemann et al. (2015) also found significant increases of scores of the Emotional Awareness scale following 3 months of bodyscan and breath awareness training, but the effect size was rather small (<0.20). Beyond the body awareness specific construct of Emotional Awareness, our findings confirm theory (Bishop et al., 2004; Phillipot and Segal, 2009) and previous evidence that suggests an association between mindfulness and general emotional awareness (Sze et al., 2010; Boden et al., 2015).

In light of evidence that demonstrates that emotional awareness is linked to better clinical outcomes and quality of life (Williams et al., 2010; Boden et al., 2015), as well as to reductions in depression symptomatology and in depression-related affective and cognitive outcomes (Goldman et al., 2005; Arch and Craske, 2006; Farb et al., 2010), the present findings of increased Emotional Awareness in the MBCT group may be relevant for clinical practice.

In the present study, the Emotional Awareness and Self-Regulation scores were strongly correlated (*r* = 0.76). Similarly, Mehling et al. (2013) found a relatively high correlation between these two scales (*r* = 0.60) in patients with chronic pain, as well as in body-mind practitioners, and Bornemann et al. (2015) also found a considerable interscale correlation between these two scales (*r* = 0.46). These findings may correspond to the notion of the general self-regulation concept (Baumeister et al., 1998; Muraven and Baumeister, 2000) as being closely related to emotion regulation (Vohs and Baumeister, 2011; Vago and Silbersweig, 2012).

### Not distracting

The significant group-by-time interaction on Not Distracting was driven by a decrease in the TAU group and an increase in the MBCT + TAU group. Distracting oneself from uncomfortable body sensations or ignoring them, is a common coping strategy in chronic pain (Reid et al., 2002; Peres and Lucchetti, 2010), as well as in depression (Matheson and Anisman, 2003). In the intervention group, however, subjects learned to pay mindful attention to all body sensations including pain and depression-related symptoms, independent of their valence. In the control group, subjects did not learn to use this alternative approach and may have practiced the more common coping strategy of distraction so that they became “better” at ignoring and distracting themselves from uncomfortable body sensations.

Correspondingly, when a sample of patients with chronic lower-back pain was compared to healthy mind-body therapy practitioners, the mind-body sample had significantly higher Not Distracting scores (Mehling et al., 2013). However, contrary to our findings, Mehling et al. (2013) found no significant difference between the Not Distracting capacities of those patients with past or current pain that did have mind-body therapy experience and those that did not have such practice. Bornemann et al. (2015) did not find that changes on the Not Distracting scale in healthy participants, following bodyscan and breath awareness training were significantly higher than those in the retest control group. These discrepancies between the studies may be due to methodological differences, differences in the interventions, and differences in the studied population. Our findings are further aligned with prior studies showing a negative association between mindfulness and experiential avoidance (Riley, 2014), and general distraction (Jain et al., 2007).

In the present study, we did not find significant correlations between the Not Distracting scale and any other MAIA scales. This result corresponds to the finding by Mehling et al. (2013) that Not Distracting scores were not correlated with the other scales in their large (*N* = 301–434) chronic pain sample. Bornemann et al. (2015) on the other hand found that the Not Distracting scale was significantly correlated with all other scales in their sample of healthy patients. This difference might be due to their very large sample size (*N* = 1076) or due to the fact that they investigated a sample of healthy participant. These findings may indicate that in patients with chronic pain, a coping style of not distracting from pain is independent of the other aspects of body awareness.

A striking finding of the present study is that Not Distracting mediates the effect of MBCT on depressive symptom reduction. This finding supports the clinical importance of the Not Distracting aspect of body awareness. Furthermore, while it has long been hypothesized that the effects of MBCT on depression are mediated by body awareness, this is to our knowledge the first study supporting such a relationship (Michalak et al., 2010, 2011, 2012; van Der Velden et al., 2015).

Although previous research has shown that for depression, distraction may be an effective coping style, mindfulness is even more effective in reducing negative mood (Broderick, 2005; Huffziger and Kuehner, 2009). Furthermore, mindfulness has been shown to be better in reducing acute pain than distraction in individuals with high levels in pain catastrophizing (Prins et al., 2014). Similarly, in patients with chronic pain that had high health anxiety, paying attention to sensations resulted in greater pain relief than distraction (Hadjistavropoulos et al., 2000), which is in line with the notion that “one problem in chronic pain is not only the pain itself, but the ‘turning away’ from, the averting of attention from the regions that give rise to painful sensations, either through deliberate distraction, or by thinking *about* the pain (conceptually) rather than experiencing the sensations directly” (Williams, 2010). More in general, experiential avoidance is associated with a wide variety of psychopathology (Hayes et al., 1996) and coping strategies that work contrary to avoidance, such as mindfulness are related to better mental health outcomes (Williams et al., 2010).

Together these findings suggest that the Not Distracting aspect of body awareness may be an important predictor of depressive symptoms and potentially of mental health in general, and that it can be cultivated through mindfulness based interventions.

### Noticing and attention regulation

The present study did not detect significant changes in the awareness of uncomfortable, comfortable, and neutral body sensations as a result of the MBCT intervention. However, the effect sizes of the pre-post changes in the treatment group were of medium size, whereas the increases in the control group only had a small effect size. These findings contrast earlier findings where large differences in MAIA Noticing scores were found between individuals with chronic pain who had mind-body practice and those patients who did not have such practice (Mehling et al., 2012). Our findings are in line with those of Bornemann et al. (2015), who despite a very large sample size did not find a significantly greater increase in the contemplative training group than in the rest control group.

No significant group by time interaction was revealed for Attention Regulation, indicating that MBCT did not result in increases of the ability to sustain and control attention to body sensations. The absence of a significant increase of Attention Regulation in the MBCT group is surprising, given that previous studies found that mind-body therapy practicing patients with back pain had higher scores of Attention Regulation than non-practicing patients (Mehling et al., 2012, 2013), and that bodyscan and breath awareness training lead to large effects on Attention Regulation in healthy participants (effect size *d* = 0.54; Bornemann et al., 2015).

It remains an open question whether the absence of significantly greater increases in Noticing and Attention Regulation in MBCT + TAU than in TAU is specific for the studied patient population and intervention, or just a lack of power. Larger studies are warranted.

### Pain catastrophizing

The absence of a group by time interaction for pain catastrophizing indicates that there were no greater changes in pain catastrophizing in the MBCT group than in the control group. However, the main effect of time was approaching significance and was driven by a large and significant decrease within the MBCT group and a small, non-significant decrease in the control group.

Consistent with the significant decrease in pain catastrophizing within the MBCT group, Mehling et al. (2013) showed that pain patients who practiced mind-body therapies worried less about their pain or other uncomfortable body sensations than individuals without mind-body practice. Furthermore, our findings are aligned with a growing body of evidence showing that in patients with chronic pain, mindfulness and participation in mindfulness-based interventions are related to decreases in pain catastrophizing (e.g., Schutze et al., 2010; Cassidy et al., 2012; Garland et al., 2012; Day et al., 2014) but see (de Boer et al., 2014).

### Limitations

The present study has several limitations. First, this pilot study had a relatively small sample size, resulting in low power to detect true effects. Yet despite this limitation, some significant effects were found. However, it must be mentioned that because of the small sample size we chose a less conservative statistical approach and did not correct for multiple comparisons. Second, because this was a pilot study, we used TAU as a control group, which does not adequately control for “placebo effects” secondary to non-specific factors like attention in patient-clinician interactions (Goyal et al., 2014). This means that, theoretically, the clinical improvement could be due to non-specific factors like attention and expectations about improvement and not due to the specific effects of MBCT. We controlled for attention as much as possible by providing equal numbers of office and phone visits with the clinician for both the MBCT group and TAU group. In future larger studies it would be imperative to compare MBCT to an active non-specific control group, such as group sessions including psycho-education and gentle stretching exercises without any mindfulness components, as in the study of Hoge et al. (2013). Third, the MAIA is a relatively new scale that requires further refinement and validation in different populations. Fourth, because the intervention produced skills and understanding of concepts that were relatively new to participants at baseline, subjects may have reached a different understanding of the concepts enquired by the MAIA items post-treatment. While the first three limitations can be addressed by larger studies with active control groups and future versions of the MAIA, the last limitation is probably inherent to interventions that result in a change of perspective and thus more difficult to address. Using behavioral data might help to validate self-report instruments in these circumstances. Finally, because there were too many missing midpoint data, we conducted mediation analysis only on the endpoint data, which decreases the degree of causal specificity (Kazdin, 2007). However, Hayes argues that mediation analysis is still valuable, even when data collection of the mediator and outcome variable is at the same time point (Hayes, 2013). The findings on the mediation analysis in this study should be interpreted as tentative and purely hypothesis generating. Future larger studies with MBCT in this population with the assessment of the MAIA scales of potential mediators at multiple time points are warranted, to permit more conclusive statements.

## Conclusion

In summary, our data suggest that MBCT can increase several dimensions of body awareness as measured with the MAIA in patients with chronic pain and comorbid active depression. In particular, the Not Distracting aspect of body awareness mediated the positive effect of MBCT on depressive symptoms. Furthermore, the MAIA appears to be a reliable instrument for measuring self-reported body awareness in this population. This finding is important because the reliability of this new and increasingly used instrument for measuring body awareness has so far not been assessed in a population of patients with chronic pain and comorbid depression. Our finding that MBCT can increase self-reported body awareness represents the first preliminary evidence in support of a causal link between a mindfulness-based intervention and increased body awareness (as measured with the MAIA) in this population. Finally, the fact that body awareness mediated the effect of MBCT on depressive symptoms provides preliminary first evidence for this long-hypothesized relationship and indicates that body awareness may have clinical relevance as an element of mindfulness approaches in the studied population.

### Author contributions

All authors listed, have made substantial, direct and intellectual contribution to the work, and approved it for publication.

### Conflict of interest statement

The authors declare that the research was conducted in the absence of any commercial or financial relationships that could be construed as a potential conflict of interest. The handling Editor declared a current collaboration with one of the authors WM on a Research Topic, though no other collaboration, and the handling editor states that the process nevertheless met the standards of a fair and objective review.
